# Preparation of High-Stability Ceramic Slurry with Gel Behavior for Stereolithography 3D Printing

**DOI:** 10.3390/ma16072816

**Published:** 2023-04-01

**Authors:** Ning Wang, Hai Chang, Chi Zhang, Yingna Wu, Rui Yang, Xing Zhang, Zirong Zhai

**Affiliations:** 1 Center for Adaptive System Engineering, ShanghaiTech University, 393 Huaxia Middle Road, Shanghai 201210, China; 2 Institute of Metal Research, Chinese Academy of Sciences, 72 Wenhua Road, Shenyang 110016, China

**Keywords:** stereolithography printing, slurry stability, rheology, loss factor, gel behavior

## Abstract

Maintaining sufficient uniformity and stability of a ceramic slurry throughout the printing cycle is key to ensuring the performance of ceramic parts in ceramic-slurry stereolithography. In this study, a stable three-dimensional network structure was introduced into a slurry to achieve slurry uniformity and stability based on rheology theory. The effects of the particle size, solid loading, dispersant content, and gelling agent content on the stability of the three-dimensional network structure in the slurry were analyzed. Relatively stable three-dimensional network structures were constructed to achieve 4-week stability of micron- and submicron-size particle slurries by adjusting the contents of the dispersant and gelling agent. Stabilization ideas for different particle size ranges are provided. In addition, an empirical stability model was obtained based on the experimental results. When the predicted empirical stability factor of *A* was less than 0.035, the slurry exhibited good stability.

## 1. Introduction

Ceramic additive manufacturing (AM) is a method of layer-by-layer production that makes it possible to create three-dimensional objects directly from digital models without machining or demolding. High design flexibility is offered, while the processing cycle and cost are decreased. One ceramic additive manufacturing technique is called ceramic stereolithography (CerSLA), which produces objects under ultraviolet (UV) light using a slurry of ceramic powder and photosensitive resin. CerSLA is renowned for its high density, high resolution, and smooth surface [1,2,3,4,5,6].

To avoid failure during debinding and sintering, the ceramic slurry must be homogeneous and stable during the printing process [4,7,8]. The major cause of the instability of a ceramic slurry is sedimentation due to gravity and flocculation owing to interparticle attraction. The former is more prevalent in a powder of large-particle slurries, whereas the latter is more critical in a colloidal solution [8]. Stokes’s sedimentation law describes the sedimentation due to gravity [9]:

(1) $$v=\frac{\left(\rho_{cerimic}-\rho_{Lliquid}\right)gd^{2}}{18\eta }.$$

Stokes’s sedimentation law still provides general guidelines for stabilizing a slurry: reduce the particle size, increase the fluid viscosity, and increase the solid fraction. This law is only applicable to dilute solutions (φ < 1 vol.%). However, Stokes’s sedimentation law is no longer applicable to colloidal solutions [10].

Overcoming the flocculation due to interparticle attraction plays a significant role in slurry stability, which mainly depends on dispersants. Dispersion mechanisms can be divided into five primary types: electrostatic stabilization, steric stabilization, electrosteric stabilization, depletion stabilization, and semisteric stabilization [8,11,12]. Zhang et al. [13] obtained a 40 vol.% solid zirconia slurry using kos110 with its steric stabilization and maintained a 96.2% stability rate after 30 days. Cai et al. [14] prepared a 50 vol.% alumina slurry using tri-ammonium citrate (TAC) and polyelectrolyte dispersant JN281. In addition, the slurry obtained by electrosteric stabilization had better dispersion and stabilization effects than the slurry obtained by electrostatic stabilization. Wiesner et al. [15] fabricated a 50 vol.% alumina aqueous slurry by electrosteric stabilization of polyvinylpyrrolidone (PVP) and finally fabricated a ceramic part with a relative density of 98%. Mario et al. [16] used MelPers 4350 with its electro-solid stabilization mechanism as a dispersant to prepare a stable 44 vol.% zirconia slurry and fabricated parts with a relative density of 99.1% and flexural strength of 741 MPa. Zürcher et al. [12] used small-molecule organic dispersants to achieve 52.5 vol.% and 55 vol.% solid loadings of submicron and micron yttria-stabilized zirconia slurries in a low-dielectric-constant organic solvent. Numerous researchers have studied five stability mechanisms to produce ceramic slurries with good performances [9,11,12,13,14,15,16,17,18,19]. However, the above studies did not provide early measurable slurry properties to judge the stability.

In theory, a stable three-dimensional network structure in a slurry under static conditions is a prerequisite for the long-term stability of dispersions or colloidal solutions, such as ceramic slurries in rheology. Under static conditions, the slurry’s storage modulus is greater than its loss modulus. A three-dimensional network structure inside a slurry can support the ceramic particles, allowing the slurry to maintain stability. As a result, the loss factor must be smaller than one under static conditions. In contrast, the slurry shows fluid properties, and the ceramic particles rapidly settle due to gravity without adequate support. However, the primer and topcoat have been the main subjects of previous investigations. This area of ceramic slurries for SLA requires further pertinent research [20,21].

Slurry stability was mainly investigated in terms of rheology in this study. Yttria-stabilized zirconia with different particle sizes was used as raw material to prepare a slurry. The requirements for building a stable three-dimensional network structure in the ceramic slurry were investigated. Meanwhile, the empirical stability formula and empirical stability factor were proposed based on the experimental results. After that, a 4-week-standing ceramic slurry was used for printing, debinding, and sintering. The surface morphology and internal defects of the sintered parts were also investigated.

## 2. Experimental

### 2.1. Materials

**Figure 1:**
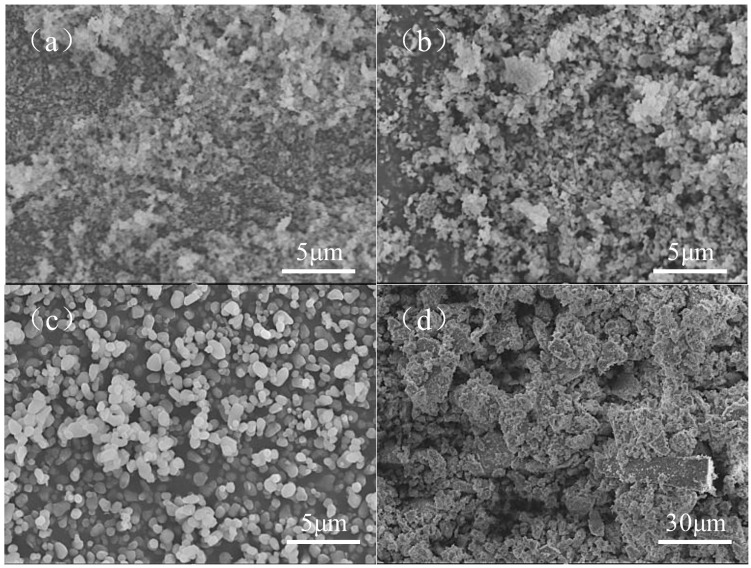
SEM images of powders with different particle sizes: (**a**) d50 = 0.21 μm, (**b**) d50 = 0.55 μm, (**c**) d50 = 1.03 μm, (**d**) 200 mesh.

Three kinds of yttria-stabilized zirconia were purchased from Shandong Sinocera Functional Materials (d50 = 0.21 µm) and Guangzhou Nanochem (d50 = 0.55 and 1.03 µm), and one kind of zirconia was purchased from Shanghai Macklin Biochemical (200 mesh) as raw materials. The particle size of powders was provided by the manufacturers. For convenience, 200, 500, and 1000 nm powder are henceforth referred to as the ZF2, ZF5, and ZF10, respectively. The detailed information of powder particle sizes of yttria-stabilized zirconia is shown in Table 1. SEM images of these powders shown in Figure 1 confirm the accuracy of powder particle size provided by the manufacturers. A mixed solution of 1,6-hexanediol diacrylate and 2,4,6-trimethylbenzoyldiphenyl phosphine oxide (TPO) was used as a photosensitive resin, purchased from Shanghai Aladdin Biochemical Technology Co., Ltd. (Shanghai, China). TEGO Dispers 655 purchased from Evonik Operations GmbH (Essen, Germany), (3-glycidyloxypropyl) trimethoxysilane (KH560) purchased from Merck KgaA (Kenilworth, NJ, USA), KOS110 purchased from Guangzhou Kangoushuang Trade (Guangzhou, China) and BYK-110, BYK-163 purchased from BYK-Chemie GmbH (Wesel, Germany) were used as dispersants. Carbomer 940, purchased from Shanghai Yuanye Bio-Technology (Shanghai, China), was used as a gelling agent.

### 2.2. Slurry Preparation

**Figure 2:**
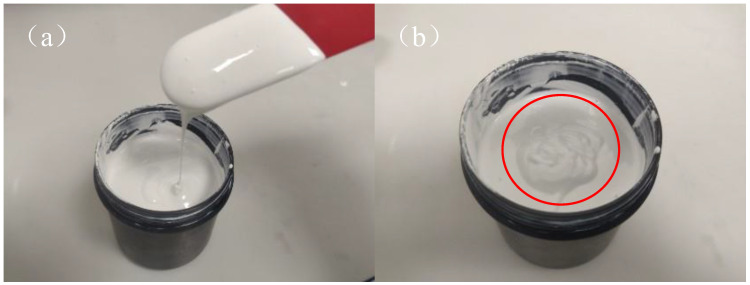
Ceramic slurry with gel behavior in (**a**) flow state, (**b**) static state.

The photosensitive resin and additives (dispersant and gelling agent) were heated in a water bath for 30 min and mixed into a clear solution. Then, zirconia powder was added to obtain the initial slurry. The slurry was milled using a planetary mill (PM1D, Road Ahead, Changsha, China) for 6 h to disperse it homogeneously. The details are shown in Table 2. The prepared slurry is shown in Figure 2, which has good fluidity and shows a similar solid state in static state. For convenience, the slurries prepared with 200, 500, and 1000 nm powder are henceforth referred to as the ZF2, ZF5, and ZF10 slurries, respectively.

### 2.3. Three-Dimensional Printing and Post-Processing

The zirconia parts were printed with an SLA printer (C100, 3DCeram Sinto, Limoges, France) equipped with a UV source with a wavelength of 405 nm using a 4-week-standing ceramic slurry. The layer thickness was set as 25 µm. The laser energy density was set to 68.57 mW/cm^2^. The printed parts were debound under an argon atmosphere with a heating rate of 1 °C/min. Then, they were heated at 1450 °C for 2 h for sintering. The sintering process was carried out under an air atmosphere.

### 2.4. Characterization

Rheological measurements were investigated by using a rotational rheometer (MCR302e, Anton-Paar, Graz, Austria) equipped with a parallel plate (PP50) and cylinder (CC17). The viscosities of the slurries were measured at 30 and 200 s^−1^ with 60 s of pre-shearing. Strain sweeps were carried out in the strain range from 0.01% to 1000% at a fixed angular frequency of 10 rad/s. The linear viscoelastic region (LVR) was confirmed by strain sweeps, using a storage modulus that deviated from the stable value by 5% as the criterion [21]. Frequency sweeps were carried out in the angular frequency range from 0.01 to 100 rad/s with fixed strain in the LVR. An angular frequency of 0.01 rad/s was simulated as a static condition.

**Figure 3:**
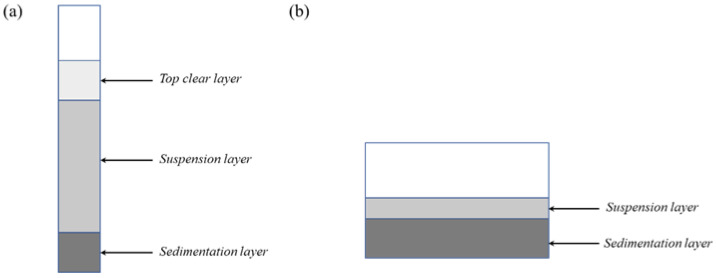
(**a**) Normal sedimentation test. (**b**) Quick sedimentation test.

Two types of sedimentation tests were performed: normal and quick sedimentation tests. First, 10 mL of the slurry was put into a test tube with a total height of 10 cm in a normal sedimentation test, as shown in Figure 3a. The stability rate was calculated by measuring the height of the top transparent layer. This method was used for the coarse powder slurries (d50 = 1.03 and 200 mesh) due to its quick sedimentation velocity. However, sedimentation always took place before the emergence of the top transparent layer, and the approach above was unable to assess the sedimentation condition appropriately [22]. In this study, the sedimentation rates of the fine powder slurries (d50 = 0.21 and 0.55 µm) were difficult to measure because no transparent top layers were visible. Thus, a quick sedimentation test was proposed to calculate the sedimentation rates of the fine powder slurries. Next, 20 mL of the slurry was put into a round container (d = 55 mm) with a total height of 7 mm in the quick sedimentation test, as shown in Figure 3b. The volume of the top flowable slurry was measured to calculate the sedimentation rate. The slurry attached to the container wall would not affect the results in this method, while the sedimentation could be judged accurately by observing the remaining slurry. To facilitate the discussion, the result of the regular sedimentation test was called the stability rate, which was then referred to as sedimentation rate in the quick sedimentation test. The formulas are as follows:

(2) $$R_{stability}=100\%\times \left(1-H_{clear}/H_{total}\right),$$

(3) $$R_{sedimentation}=100\%\times \left(1-V_{flowable}/V_{total}\right),$$

where *R_sedimentation_* and *R_stability_* are the sedimentation rate and stability rate, respectively, *H_total_* and *V_total_* are the total height and volume of slurry, respectively, *H_clear_* is the height of the top clear part, and *V_flowable_* is the volume of flowable slurry.

Thermogravimetric analysis (TGA) was conducted to ascertain the debinding curve (STA 449 F3 Jupiter, NETZSCH, Selb, Germany). The measurement was performed at a heating rate of 5 °C/min from 50 to 800 °C in an argon atmosphere. Moreover, linear shrinkage was carried out to ascertain the sintering curve (DIL 402 Expedis Classic, NETZSCH, Germany). The measurement was conducted with a heating rate of 5 °C/min from 50 to 1600 °C in an air atmosphere.

The slurry agglomeration was tested with a scraper fineness meter ranging from 0 to 50 µm. The surface morphologies of the sintered parts were investigated using a laser confocal microscope (LSM 900, Zeiss, Oberkochen, Germany) with a 1.5 µm slice thickness. The scanning area was 2 × 2 mm. X-ray imaging microscopy was performed using a three-dimensional X-ray microscope (Xradia 620 Versa, Zeiss, Germany) operating at an accelerating voltage of 160 kV and power of 25 W. For a 1.35 µm pixel resolution data, 1201 projection images were obtained over 180°, each with an exposure time of 3 s. The three-dimensional renderings presented here were created using the commercial software Dragonfly Pro ver.2019. The pore segmentation was processed with a deep learning module within Dragonfly Pro ver.2019.

## 3. Results

### 3.1. Effects of Dispersant Species and Content on Slurry Viscosity

Due to the hydroxyl groups on their surfaces, ceramic powders are hydrophilic and are weakly compatible with resin, which make them challenging to distribute uniformly in an organic system. Dispersants can reduce the hydrophilicity of ceramic powders and raise the solid loading of ceramic slurries. It is essential to evaluate how the dispersant type and content impact the fluid properties and stability of the ceramic slurry [19].

**Figure 4:**
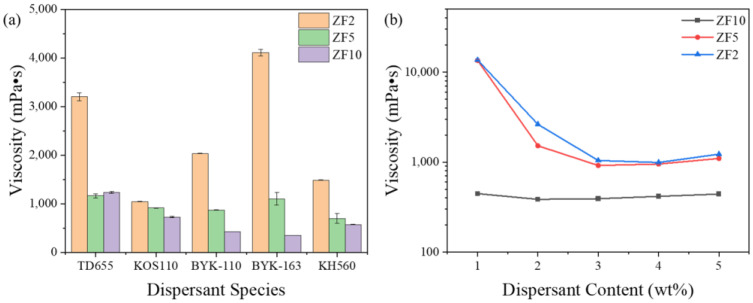
Effects of (**a**) dispersant species and (**b**) dispersant content on slurry viscosity.

Figure 4a shows the viscosity of the slurry with different particle sizes under different dispersants (3 wt% of powder). The results indicate that the dispersants applicable for the different zirconia powders were not the same. The optimal species were KOS110 with a viscosity of 1042.19 mPa•s for the ZF2 slurry (40 vol.%), KH560 with a viscosity of 697.26 mPa•s for the ZF5 slurry (40 vol.%), and BYK-163 with a viscosity of 346.95 mPa•s for the ZF10 slurry (47 vol.%). The adsorption efficiency of the dispersant on the surface of the ceramic powder was thought to be affected by the various surface characteristics of the powders as a result of factors such as the fabrication process and storage. This also indicates that a general dispersant was difficult to obtain for most ceramic powders.

Moreover, Figure 4a shows a decreasing trend of the viscosity with increasing particle size, which has been reported by Liu et al. [23]. The inverse relationship between the particle size and specific surface area was the primary contributor to this phenomenon. A high specific surface area increased the powder resistance when flowing into the resin. The dispersant species should ideally be KOS110 to facilitate the discussion of the results.

The dispersant content was positively correlated with the powder-specific surface area and optimal dosage. To determine the optimal dispersant dosage for different powders, the viscosities of the slurries prepared with 1 to 5 wt% KOS110 are shown in Figure 4b. It was observed that the viscosity of the slurries decreased with increasing dispersant content, which was 2 wt% for the ZF10 slurry, 3 wt% for the ZF5 slurry, and 4 wt% for the ZF2 slurry. The mechanism of KOS110 was steric stabilization. When the content was lower than the optimal value, the adsorption quantity of powder was insufficient, resulting in inadequate dispersion of the powder and high viscosity of the slurry. However, when the content was higher than optimal, the excess KOS110 was free inside the resin, bridging between particles due to its long-chain molecules, and resulting in a viscosity increase [24,25].

### 3.2. Effects of Particle Size on Loss Factor

**Figure 5:**
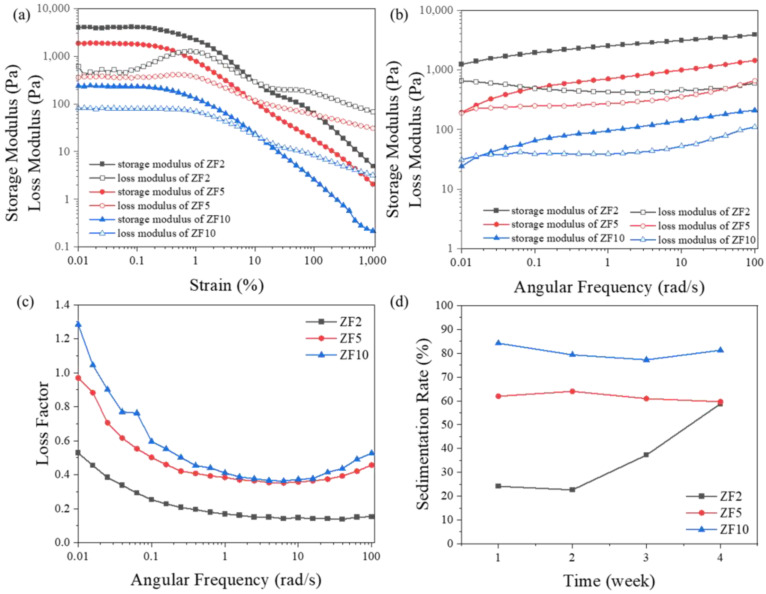
Effects of particle sizes on modulus under various (**a**) strains and (**b**) angular frequencies, (**c**) loss factor under angular frequency and (**d**) stability.

As mentioned earlier, slurry stability in rheology requires a stable internal three-dimensional network structure when static. It has been reported that a powder can form a three-dimensional network structure inside a slurry, showing a solid-like response [8]. To investigate the conditions for creating stable three-dimensional network structures in a slurry under static conditions, slurries with different particle sizes (45 vol.%) were prepared, and the rheological behaviors were examined. Figure 5a shows the modulus values for different particle sizes of the slurry under strain sweeps. This was used to determine the LVR. The end of the LVR was slightly higher than a 0.1% strain. Above 0.1% strain, the slurry began to yield. The storage modulus increased with the decrease in the particle size, which indicated that the three-dimensional network structure inside the slurry became stronger and stronger. This proved the negative correlation between the particle attraction tendency and the particle size. The slurry yielded at a lower strain with a high internal structural strength. This was comparable to the relationship between a solid’s strength and plasticity, wherein a higher strength is accompanied by worse resistance to deformation [26]. Figure 5a also shows the peak of the loss modulus, which increased with the decrease in particle size. This peak indicated the damage process of the slurry microstructure. The microcracks grew into macrocracks with an increase in tension.

The loss modulus was increased because of the energy loss brought on by crack initiation and propagation when the strain increased past the LVR. When the strain was higher than the peak strain of the loss modulus, the internal structure of the slurry was destroyed, and then, the loss modulus decreased. The large-particle-size slurry exhibited a more fluid-like behavior. The strength of the interior three-dimensional network structure was low, and it was difficult to see how cracks initiated and propagated. Additionally, this obscured the peak value of the loss moduli of the slurries with large particle sizes [21].

The moduli and loss factors of the slurries with different particle sizes under frequency sweeps are shown in Figure 5b and Figure 5c, respectively. The test strain was limited to the LVR to ensure that the microstructure was not damaged. In rheology theory, when the storage modulus is higher than the loss modulus, that is, the loss factor is less than 1, the changes of the two are similar and they non-intersect, and the suspension remains stable for a long time [20,21]. However, the result showed an intersection trend between them. The loss factor must be higher than 1 at very low frequencies, which indicated the inevitability of sedimentation. However, the growth rate of the loss factor could still be used to evaluate the relative stability. The loss factor under the simulated static conditions (ω = 0.01 rad/s) is shown in Figure 5c, showing a relatively stable three-dimensional structure inside the ZF2 and ZF5 slurries with tanδ = 0.528 and 0.970, respectively. However, the structures of the ZF5 slurry failed quickly with a further frequency reduction. Theoretically, it is challenging to classify the ZF5 slurry as a stable slurry.

For the ZF10 slurry, there was no stable internal three-dimensional structure with tanδ = 1.284, meaning that fast sedimentation occurred. This was consistent with the 4-week sedimentation rate shown in Figure 5d. The ZF2 slurry was maintained for 2 weeks without sedimentation due to the relatively stable three-dimensional structure. However, the ZF5 and ZF10 slurries were stable for less than 1 week. Sedimentation was noted within 24 h after they were prepared. The sedimentation result verified the feasibility of using the loss factor to evaluate the stability of the slurry.

### 3.3. Effects of Solid Loading on Loss Factor

**Figure 6:**
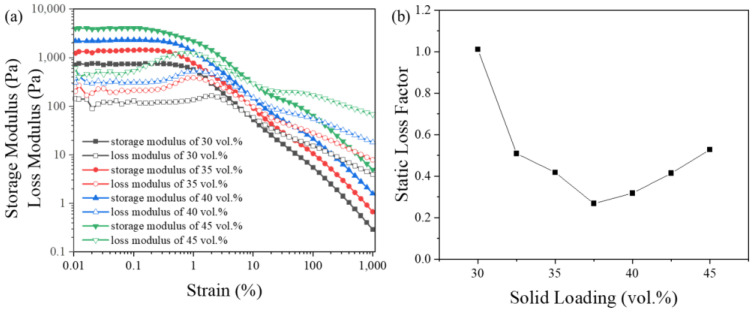
Effects of solid loading of ZF2 slurries on (**a**) modulus under various strain and (**b**) static loss factor.

The three-dimensional structure of the slurry depends on the interactions between particles, which means that the solid loading could affect the loss factor. The ZF2 slurry with a solid loading of 30–45 vol.% was prepared to evaluate the structure inside. As shown in Figure 6a, the modulus was positively correlated with the solid loading. The higher the solid loading was, the more opportunities there were for interactions between particles to occur, and the greater the strength of the microstructure. The range of the LVR showed a negative correlation with the solid loading. The reason was the same as that for the negative correlation between the particle size and LVR range, which was explained above.

The loss factor under static conditions was measured at a fixed angular frequency of 0.01 rad/s. The strain was confined to the linear viscoelastic region. The results in Figure 6b show a relatively stable three-dimensional structure in the slurry under a simulated static state when the solid loading was higher than 30 vol.%. The most stable three-dimensional structure in the slurry appeared to be a 37.5 vol.% slurry loading. The loss factor showed an upward trend with a further increase in the solid loading. It is speculated that this was related to interparticle flocculation. The increase in the solid loading aggravated the interparticle flocculation tendency, resulting in large internal particles. This weakened the stability of the three-dimensional structure inside. This also revealed the two sides of the three-dimensional structure inside the slurry under general conditions. The three-dimensional structure, although preventing the slurry from sedimentation, also created conditions for the agglomeration of particles, which caused sedimentation. It has been reported that the solid loading of the slurry used in stereolithography (SLA) should be higher than 40 vol.% to ensure a high sintering density [8]. This means that a three-dimensional structure was spontaneously generated inside the ZF2 slurry, showing relative stability under static conditions.

### 3.4. Effects of Dispersant Content on Loss Factor

**Figure 7:**
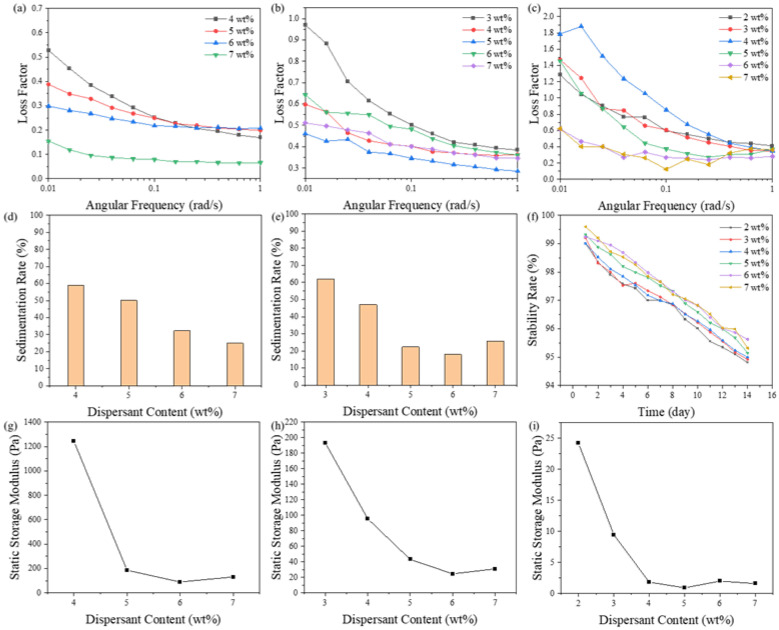
The effects of dispersant content on loss factor under various angular frequency in (**a**) ZF2, (**b**) ZF5, (**c**) ZF10 slurries, stability of (**d**) ZF2, (**e**) ZF5, (**f**) ZF10 slurries, and static storage modulus of (**g**) ZF2, (**h**) ZF5, (**i**) ZF10 slurries.

It has been reported that the increased dispersant content can strengthen the connection between particles, causing an increase in the slurry viscosity [24,25]. This suggested that it might be possible to enhance the slurry’s static three-dimensional structure by employing excessive dispersant. Figure 7 shows the relationship between the loss factor, stability, and storage modulus of slurries with various dispersion contents. The rising tendency of the loss factor was decreased by the addition of dispersants, as illustrated in Figure 7a–c. Due to the excessive dispersant utilized, the three-dimensional structure’s stability was improved by providing bridges between the particles. Excessive dispersant improved the slurry’s stability, as shown in Figure 7d–f. For the ZF2 and ZF5 slurries, the 4-week sedimentation rates dropped from 58.75% to 25.03% and from 61.93% to 25.71%, respectively. The 2-week stability rate increased from 94.81% to 95.32%. Furthermore, 20%–30% sedimentation was observed in a 4-week sedimentation test when the dispersant content was higher than 6% in the ZF2 slurry and 5% in the ZF5 slurry. However, this was due to the low statistics of flowable volume caused by some slurry adhering to the container wall and bottom. The bottoms of these five groups had no un-flowable parts, meaning that there was no settling. To prevent direct contact between the particles, the extra dispersant built a bridge between them. This also prevented agglomeration from occurring. The sedimentation experiment showed that for the ZF2 and ZF5 slurries, 1.5 times the recommended amount of dispersant efficiently prevented sedimentation in 4 weeks. The loss factor of the ZF10 slurry also had a negative relationship with the dispersant content. When the dispersant content was higher than 6%, the loss factor was lower than 1. A reasonably stable three-dimensional network structure was eventually established when the ZF10 slurry was in a static state.

Moreover, the stability rate data showed that the stability was positively impacted by the rise in dispersant content. However, all the ZF10 slurry groups exhibited settling within two days. The rise in dispersant content decreased the slurry’s storage modulus, as shown in Figure 7f–i. The storage modulus was only 1.61 Pa in the ZF10 slurry with a 7% dispersant content, meaning that the three-dimensional structure strength of the slurry was very weak. At the same time, there was a negative correlation between the strength of the three-dimensional structure in the slurry and the particle size. However, the weights of the particles were proportional to the third power of their sizes. The three-dimensional structure strength in the slurry could not support the weight of the particles with the increase in the particle size and dispersant content, resulting in the rapid sedimentation of the ZF10 slurry. This showed that the three-dimensional structure in the slurry required a specific structural strength to ensure the anti-settling characteristics of the slurry.

The results in Figure 5a show that the reduction of the particle size greatly increased the strength of the three-dimensional network structure inside the slurry. They also implied that there were still significant bonds between the small ceramic particle powders in the slurry, even at the optimal dispersion concentration. The close interactions made it simple for ceramic particles to coagulate, which disrupted the three-dimensional structure. When the slurry contained excessive dispersant, dispersant bridging replaced the connections between particles. The strength of the three-dimensional network structure in the slurry was negatively correlated with the dispersant concentration because the bridging strength was weaker than the direct interaction of the powder.

The three-dimensional structure in the slurry was more stable because the dispersant’s bridging prevented the powder from directly contacting it. It also demonstrated that the effect of maintaining slurry stability by modifying the dispersant content was more apparent for fine powder and may not be beneficial for large particle sizes.

### 3.5. Effects of Dispersant Content on Curing Depth and Viscosity

**Figure 8:**
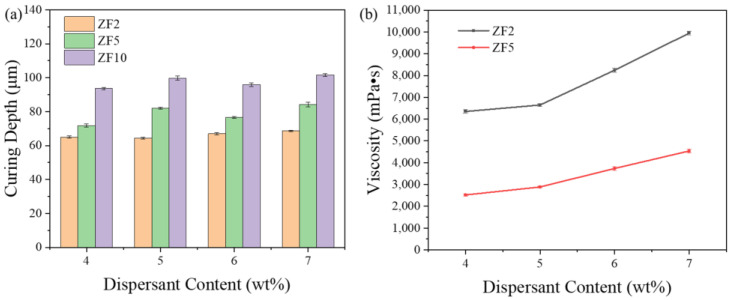
(**a**) Effects of the dispersant content on curing depth and (**b**) the relationships between the viscosity and dispersant content at printing conditions.

As the dispersion content increased, the photosensitive resin per unit volume dropped. For instance, in this experiment, the dispersant concentration was increased from 4% to 7%, resulting in an 8.24% rise in the proportion of dispersant in the unit volume slurry. This appeared to have an impact on the slurry’s curing ability. In Figure 8a, the curability of a slurry with a dispersant level of 4–7% and three particle sizes were studied. The findings demonstrated that an increased dispersant content did not affect the curing thickness significantly. Instead, the particle size had a significant impact on the curing thickness. In summary, there was a positive correlation between the particle size and curing thickness, which has been explained by the curing formula proposed by Griffith [27]:

(4) $$D_{c}=\frac{2}{3}\cdot \frac{dn_{0}^{2}}{\varphi \tilde{Q}\Delta n^{2}}\ln\frac{E_{0}}{E_{C}}.$$

Thus, there was a linear relationship between the curing thickness and particle size. The curing results in this study were consistent with the theoretical results.

The viscosity changes with the dispersant content at the printing shear rate are shown in Figure 8b. In this study, the printing shear rate was 200 s^−1^. Because the stabilization method of the ZF10 slurry was different from that of ZF2 and ZF5, only the viscosity changes of the ZF2 and ZF5 slurries are shown here. As expected, the viscosity was positively correlated with the dispersant content. The excess dispersant was free inside the resin. Due to the long-chain molecules of the dispersant itself, a bridge was formed between the powder particles, resulting in a continuous increase in the viscosity. The curing and viscosity tests revealed that increasing the dispersant content would stabilize the slurry primarily but had no meaningful influence on the curing thickness. This allowed it to use more stable coating parameters, resulting in less printing part damage throughout the printing process.

### 3.6. Effects of Gelling Agent Content on Loss Factor

In addition to depending on the particle interconnections, the three-dimensional structure in the slurry was generated by the resin itself, which was achieved by adding a gelling agent. The gelling agent was a polymer with a high molecular weight that formed a three-dimensional network structure in solution via polymer entanglement. Dou et al. used carbomer 940 and HE-cellulose to build a three-dimensional network inside the slurry under static conditions and achieved ceramic stereolithography printing under microgravity [28]. Referring to Dou’s work, carbomer 940 was used as a gelling agent to study the effect of slurry stability.

**Figure 9:**
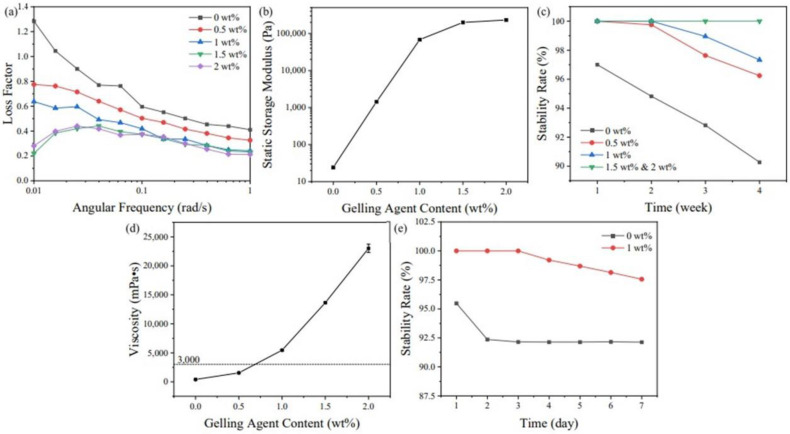
(**a**) The relationship of angular frequency and loss factor under various gelling agent content, and the effects of gelling agent content on (**b**) static storage modulus, (**c**) stability rate, (**d**) viscosity of ZF10 slurries, and (**e**) stability rate of 200-mesh slurries.

Figure 9a,b show the variations of the loss factor and static storage modulus of the ZF10 slurry with different gelling agent contents. The results showed that increasing the content of gelling agent could significantly reduce the loss factor and increase the storage modulus. The storage modulus increased from 24.21 to 1436 Pa with the addition of 0.5% of the gelling agent, indicating that the three-dimensional structure of the slurry was strengthened. The simulated static loss factor decreased from 1.284 to 0.281, eliminating the rising trend in the very-low-frequency region. Therefore, there was a solid three-dimensional structure in the slurry in the static state, showing long-term stability. The stability rate results are shown in Figure 9c. The solid three-dimensional structure effectively prevented the settling, and settling was observed the next day without a gelling agent. When the content was higher than 1.5%, the slurry did not settle after 4 weeks. However, due to the mechanism of the gelling agent, a solid three-dimensional network structure was formed inside the slurry, which could increase the viscosity of the slurry. Figure 9d shows the relationship between the gelling agent content and viscosity.

When the content of the gelling agent was increased from 0% to 2%, the viscosity increased significantly from 405.92 to 23,013.51 mPa•s at 30 s^−1^. A high-viscosity slurry had difficulty self-leveling, which makes the slurry with added gelling agent more suitable for sinking printers equipped with scrapers, limiting the available printer models. At the same time, more conservative recoating parameters should be required to reduce damage to the printing parts. However, Figure 9c,d also show that adding 0.5% of the gelling agent could result in stability for seven days. The viscosity only increased to 1543.55 mPa•s at 30 s^−1^, which was lower than the generally accepted viscosity requirement of 3000 mPa•s [8]. Thus, the applicability of this slurry to a broader range of printers can be improved by adding a gelling agent based on the expected printing time of the parts. Small parts can use low gelling agent contents to obtain a lower slurry viscosity. For large parts with long printing times, high content of gelling agents can be used to ensure the reliability of the parts.

To further verify the anti-settling effect of the gelling agent, a larger particle size of zirconia (200 mesh) was configured as a slurry with a 1% gelling agent to test the stability rate. The results are shown in Figure 9e. The slurry without a gelling agent settled completely in two days, while a 1% content of gelling agent could maintain stability for about three days. This showed the positive role of the gelling agent in preserving stability and provides a method to achieve stability of large-particle-size and overweight powder slurries.

### 3.7. Post-Processing

Three slurries with different particle sizes were used for printing after standing for 4 weeks, and their components are shown in Table 3. The printing parameters were a layer thickness of 25 μm and a laser output power of 68.57 mW/cm^2^. Flake (10 × 10 × 3 mm) and cylindrical (Φ 5 × 12 mm) samples were printed for TGA and dilatometry (DIL) tests, respectively.

**Figure 10:**
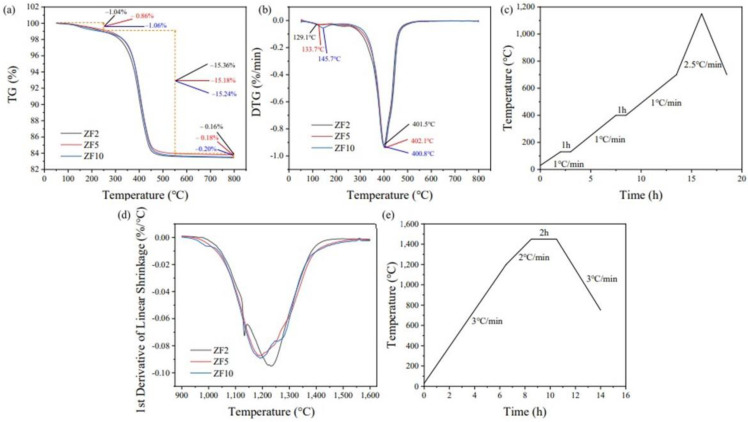
The (**a**) thermogravimetry (TG) curves, (**b**) derivative thermogravimetry (DTG) curves and (**c**) the detail debinding curves of the green parts with various particle sizes, and (**d**) the first-derivative of linear shrinkage in the Z direction and (**e**) detail sintering curve of the parts with various particle sizes.

The organic component content in the green body printed in this study was about 55 vol.%. It is necessary to determine the appropriate debinding process to obtain reliable parts. Figure 10a,b show the thermogravimetry (TG) and derivative thermogravimetry (DTG) curves in an argon atmosphere. The results showed no significant difference in the debinding of the green body formed using the three kinds of slurries. Two main stages of weight loss were observed. The first stage was from room temperature to 250 °C, in which mainly green body evaporation of small-molecule components and cleaning agents occurred [29]. The second stage was from 250 to 550 °C. This section was the pyrolysis temperature range of the cured resin, and the peak pyrolysis temperature was about 400 °C. According to the TGA test results, the debinding curve was designed as shown in Figure 10c. In addition, the weight losses of the three groups were about 1 wt% lower than the theoretical weight loss value for the green body. The samples appeared black after the TGA tests, which was considered to be due to carbon residue in the green bodies. It is presumed that this was due to the lack of oxygen participation in the debinding process, resulting in incomplete pyrolysis of the resin. This part of the carbon was removed during sintering.

Figure 10d shows the printed part’s DIL test results in the Z-direction. The results showed that the sintering of the 3Y zirconia printed parts began at about 1000 °C. The peak contraction occurred at about 1200 °C. At around 1450 °C, the shrinkage rate was less than 0.05%/min when the sintering process was complete. There was an apparent offset and an abnormal shrinkage peak of the ZF2 parts compared with the results of the other two groups. Based on the appearance of the sample after the test, it is presumed that this was due to the rapid heating rate resulting in a sudden slight cracking of the sample during the test, affecting the test result. This indicates that the heating rate of 5 °C/min may have been too fast. Therefore, the sintering curve in Figure 10e was designed.

### 3.8. Roughness and Defects

**Figure 11:**
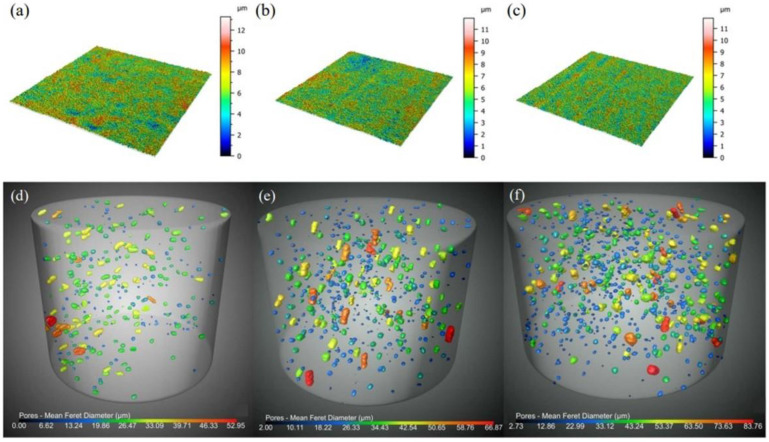
The surface morphologies of (**a**) ZF2, (**b**) ZF5 and (**c**) ZF10 sintered parts, and the computed tomography (CT) results of (**d**) ZF2, (**e**) ZF5 and (**f**) ZF10 sintered parts. (The scales in figures (**d**–**f**) are only applicable to micropores).

Figure 11a–c show the surface morphologies of the sintered parts made from slurries with different particle sizes. Only a few protrusions were detected on the parts’ surfaces of about 4 mm^2^, meaning that the flatness was good. No agglomeration was observed in the slurry after standing for 4 weeks. The surface roughness values of the ZF2, ZF5, and ZF10 parts were 4.083, 3.773, and 2.775 μm, respectively. The roughness was higher in the small-particle-size parts due to the higher agglomeration tendency between the powders in the small-particle-size slurry. After debinding and sintering, the surface fluctuations of the components were worse due to coagulation between tiny particles. However, only a small number of particles were involved in this coagulation. This did not imply that the slurry experienced wide-scale agglomeration and produced large particle aggregates.

The computed tomography (CT) results of a slurry with various particle sizes are displayed in Figure 11d,e. The CT results showed that the internal defects mainly existed in the form of micropores. To make the defects easier to observe, the micropores in Figure 11d,e were magnified. The rulers in Figure 11d,e were only applicable to micropores. The ZF2, ZF5, and ZF10 samples achieved sintering consistencies of 99.67%, 99.29%, and 98.90%, respectively. This demonstrated that the slurry’s internal three-dimensional network structure had no noticeable impact on the printing quality. However, the samples with different particle sizes showed a positive correlation between the particle size and porosity. In solid-state sintering, the driving force of ceramic densification came from the reduction of free energy. This meant that the smaller the particle size was, the greater the densification driving force was, which made the densification rate inversely proportional to the particle size [30]. All groups of samples were heat treated at 1450 °C for 2 h. Under this process condition, the densities of the ZF5 and ZF10 samples could still be further improved. Prolonging the holding time could increase the sintering densities of ZF5 and ZF10.

## 4. Discussion

### 4.1. Evolution of Structure inside the Slurry

In rheology, the stability of a slurry depends on a stable three-dimensional structure inside. Four factors were pointed out earlier to affect the stability of the three-dimensional structure of slurry: particle size, solid loading, dispersant content, and gelling agent content. The evolution of the three-dimensional structure inside the slurry under different factors is further discussed.

#### 4.1.1. Particle Size

**Figure 12:**
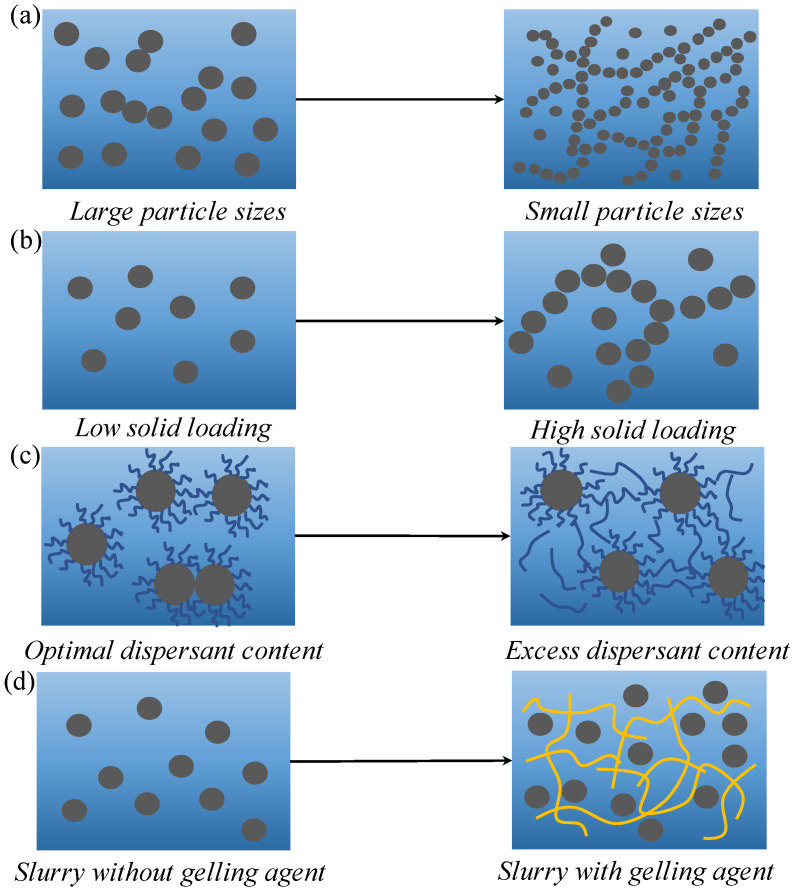
The evolution of the structure inside slurry with the varieties of (**a**) particle size, (**b)** solid loading, (**c**) dispersant content and (**d**) gelling agent content.

Figure 12a shows the variation of the static three-dimensional structures of the slurries with particle sizes. As noted earlier, the static loss factor of the large-particle-size slurry was >1, meaning that there was no stable three-dimensional structure inside the slurry to resist powder settling. The large-particle-size powder’s specific surface area was insufficient to provide an efficient connection between the particles, which caused the slurry to exhibit more fluid characteristics and settle more rapidly. The lower the particle size was, the lower the static loss factor value of the slurry was. Stronger bonds between powder particles would result from more opportunities for contact as the specific surface areas of the particles increased. The internal crosslinking of the slurry was strengthened, with a relatively stable three-dimensional structure to maintain the stability of the slurry. However, the three-dimensional structure in the slurry was strengthened at a small particle size, and the flocculation tendency was also improved, as shown by the surface roughness of the printed parts noted earlier.

#### 4.1.2. Solid Loading

Figure 12b shows the variation of a three-dimensional structure with solid loading in a slurry under static conditions. As noted earlier, the static loss factor showed a U-shaped change. There were no effective connections between the powder particles inside the slurry at low solid loading. The increase in solid loading strengthened the connections between particles, and the internal crosslinking degree of slurry also gradually increased. At 37.5 vol.%, the static three-dimensional structure of the slurry was the strongest. However, the flocculation tendency between the particles increased at a higher solid loading, leading to powder aggregates in the slurry. The agglomerates weakened the slurry’s three-dimensional stability and gradually increased the static loss factor by acting as large particles. As the solid loading increased, the slurry began to exhibit fluid characteristics. However, due to the dispersant, agglomerates only accounted for a small number of the particles. The three-dimensional structure in the slurry was still relatively stable. The slurry primarily showed solid characteristics in static conditions, maintaining its relative stability.

#### 4.1.3. Dispersant Content

Figure 12c shows the change in the three-dimensional structure of slurry with the dispersant content under static conditions. Figure 7a–c show that increased dispersant content could slow the loss factor rise and reduce the static loss factor. This indicated that the addition of dispersant could enhance the degree of cross-linking and gradually form a relatively stable three-dimensional structure in the slurry. The dispersant used in this study was a long-chain polymer. When the content was higher than the optimal content, the redundant dispersant molecules were freely dispersed in the resin, and the long-chain structure of the dispersant itself formed bridges between particles, improving the internal crosslinking degree of slurry and forming a more stable three-dimensional structure. Table 4 shows the agglomeration of the ZF2 slurry with different dispersant contents. Samples were taken from flowable parts of the slurry after standing for 4 weeks. At the optimal content of 4%, there were approximately 12 μm large agglomerated particles in the slurry, while at a content of 7%, there were no noticeable agglomerated particles. This resulted from the excessive dispersant forming a bridge between the particles. Even so, the direct contact of the particles and their agglomeration was hindered. This showed that excessive dispersant had strong advantages in terms of slurry stability and anti-agglomeration.

#### 4.1.4. Gelling Agent Content

As shown in Figure 12d, the three-dimensional structure of the slurry changed under static conditions as the gelling agent increased. A high-molecular-weight polymer known as a gelling agent could form a three-dimensional structure by polymer entanglement in solution. Unlike other factors, the gelling agent generated a relatively stable three-dimensional structure by entangling its molecular chains in the slurry. The increase in the gelling agent content gradually strengthened the crosslinking inside the slurry, and the powder particles were wrapped in the holes formed by polymer chains. The polymer network created by the gelling agent provided strong support for the particles. This strength support resulted in the large-particle-size and overweight powder slurry containing the gelling agent remaining stable for a period.

### 4.2. Empirical Stability Model

Figure 7a–c show a significant correlation between the variation rate of the loss factor in the low-frequency region (0.01–1 rad/s) and the slurry stability. However, there is no relevant model to describe the relationship between the two. An empirical model between the two is established based on the results of this study, and the empirical stability factor is provided to judge the strength of the slurry stability quickly.

Many mathematical models can describe the loss factor of a slurry in low-frequency regions showing an upward trend. However, considering the decreasing trend of the loss factor in Figure 9a, a quadratic function is a simple and feasible model. The value of the quadratic term coefficient can be used to describe the variation rate of the loss factor. To simplify the mathematical model, the logarithms of the loss factor values in the low-frequency region were used. The fitting model is as follows:

(5) $$T=A{\left(-\mathrm{lg}\omega \right)}^{2}+B\left(-\mathrm{lg}\omega \right)+C,$$

where *T* is the loss factor value (tan δ), *ω* is the angular frequency, and the quadratic term coefficient *A* is named the empirical stability factor. The fitting model is named the practical stability model. The fitted data are shown in Table 5.

Table 5 confirms the negative correlation between the variation rate of the loss factor in the low-frequency region and the slurry stability. This is shown by the empirical stability factor *A*. Except when the gelling agent content was higher than 1%, the R^2^ values for most groups of data and the fitting model were above 0.96, showing the excellent applicability of the fitting model. Although the correlation of the groups with a gelling agent content higher than 1% was insufficient, the loss factors of the two groups showed a downward trend in the extremely low-frequency region. When the loss factors did not rise in the low-frequency region, stable three-dimensional structures were created inside the slurry in the static state. Compared with the specific value of the empirical stability factor *A*, the plus–minus of the empirical stability factor *A* can better show the stable state of slurry with a gelling agent. In contrast, the R^2^ value is not very important.

The data in Table 5 are summarized to clarify the effect of the empirical stability factor *A* on the stability of the slurry. The results of the slurry without a gelling agent show that the empirical stability factor *A* of the groups reaching 4-week stability had a maximum value of 0.0335, and the groups in which sedimentation occurred in 4 weeks had a minimum value of 0.0456. This shows that the value of empirical stability factor *A* can be used to judge the slurry stability. For a slurry without a gelling agent, 0.035 can be selected as the judgment basis for the relative stability of the slurry. For a slurry containing a gelling agent, the empirical stability factor *A* of the 4-week stability groups reached a negative value. The empirical stability factor *A* of the group with 1% gelling agent was 0.0264, but the stability of this group was only maintained for at least 2 weeks. It is presumed that this was due to the different construction methods of the internal three-dimensional structures of the two slurries. However, 2 weeks is sufficient for printing most parts. The empirical stability factor value of 0.035 can be considered to be a suitable assessment value for slurry stability. To acquire the slurry’s long-term storage stability, it is advised that the empirical stability factor *A* be changed to a negative value for the slurry containing a gelling agent.

At present, the slurry stability test mainly relies on settling tests. These experiments often take several weeks [9,13,17,18,24,31,32,33]. However, the time required for measuring the loss factor is about one hour, and the time required to test slurry stability can be significantly reduced. The empirical stability model provides a fast, indirect measurement method for slurry stability. In addition, the empirical stability model in this study is based on the prerequisite that the static loss factor is less than 1, which was determined by rheological theory. Once the static loss factor is greater than 1, the slurry behaves as a fluid in a static state, and there is no relatively stable three-dimensional structure inside. At this time, regardless of the empirical stability factor value, the slurry settles rapidly. At the same time, it was pointed out earlier that the three-dimensional structure of a slurry requires a certain strength, and the general value of this strength was presumed to be 10 Pa in this paper, that is, the structure must reach a relatively strong gel state [21]. The strength must be improved for a large-particle-size and overweight powder slurry. Further research is needed to determine the specific increase range.

## 5. Conclusions

The slurry stabilization technique presented in this study was based on rheology principles. Additionally, we evaluated how the stability was affected by particle size, dispersion content, solid loading, and gelling agent content. The results demonstrated that the slurry’s three-dimensional network structure successfully prevented settling. The results of settling tests demonstrated that no settling occurred in the slurry. The printing and sintering findings also showed that the slurry’s internal three-dimensional network structure had no discernible influence on the quality of printing or sintering. Furthermore, the following conclusions were obtained.

(1) The particle size was negatively correlated with the stability of the internal three-dimensional network structure of the slurry. The 200 nm powder slurry could form a stable three-dimensional network structure at a 45 vol.% solid loading.
(2) A U-shaped curve relating the stability of the three-dimensional network structure in the slurry and the solid loading was established. To create a three-dimensional network structure, the solid loading of a 200 nm slurry should be larger than 32.5 vol.% at least. Three-dimensional structure networks have the highest stability at 37.5 vol.%. Due to powder agglomeration, the stability of the slurry is compromised at larger solid contents.
(3) At 1.5 times the ideal dispersant concentration, the slurry’s stability might last for 4 weeks. However, when the dispersant level increased, the strength of the three-dimensional network structure decreased, making the technique only effective for slurries with small particle sizes.
(4) The addition of a gelling agent could significantly improve the stability and strength of the three-dimensional network structure in the slurry. For large-particle-size powder slurries, a gelling agent could be added to build a stable three-dimensional network structure to maintain slurry stability. However, the relationship between the slurry viscosity and gelling agent content must be balanced to ensure a smooth recoating process.
(5) The experimental results provided the empirical stability model and the empirical stability factor *A*. When *A* is less than 0.035, the slurry is stable. The robust slurry stability test can be completed in less than 1 h.

## Figures and Tables

**Table 1:** Detailed particle size distributions of yttria-stabilized zirconia.

	d10 (μm)	d50 (μm)	d90 (μm)
ZF2	0.14	0.21	0.50
ZF5	0.26	0.55	0.92
ZF10	0.59	1.03	1.83

**Table 2:** Slurry composition.

Materials	Contents
Zirconia powder	30–45 vol.% of slurry
Dispersant	1–7 wt% of Zirconia powder
Gelling agent	0–2 wt% of slurry
Photosensitive resin	bal.

**Table 3:** Slurry composition for printing.

	Solid Loading (vol.%)	Particle Sizes (μm)	Dispersant Content (wt%)	Gelling Agent Content (wt%)
ZF2	45	200	6	0
ZF5	45	500	5	0
ZF10	45	1000	2	1.5

**Table 4:** Agglomerate sizes of ZF2 slurry.

Dispersant Content (wt%)	4	5	6	7
Agglomeration size (μm)	~12	~7	not evident	not evident

**Table 5:** Fitting data statistics.

	Dispersant Content (wt%)	Gelling Agent Content (wt%)	Empirical Stability Factor *A*	R^2^	Stable Time (Day)
**ZF2**	4	0	0.0930	0.9978	~14
**ZF2**	5	0	0.0456	0.9975	~14
**ZF2**	6	0	0.0310	0.9916	>28
**ZF2**	7	0	0.0335	0.9430	>28
**ZF5**	3	0	0.1892	0.9911	<1
**ZF5**	4	0	0.0936	0.9690	<7
**ZF5**	5	0	0.0253	0.9775	>28
**ZF5**	6	0	0.0227	0.9745	>28
**ZF5**	7	0	0.0256	0.9787	>28
**ZF10**	2	0	0.2200	0.9865	<1
**ZF10**	3	0	0.3248	0.9790	<1
**ZF10**	4	0	0.2591	0.9775	<1
**ZF10**	5	0	0.5350	0.9922	<1
**ZF10**	6	0	0.1786	0.9226	<1
**ZF10**	7	0	0.2897	0.8453	<1
**ZF10**	2	0.5	0.0387	0.9883	~7
**ZF10**	2	1	0.0264	0.9781	~14
**ZF10**	2	1.5	−0.1436	0.6861	>28
**ZF10**	2	2	−0.1212	0.8123	>28

## Data Availability

Data will be made available on request.
